# Comparative transcriptome analysis of genes involved in the drought stress response of two peanut (*Arachis hypogaea* L.) varieties

**DOI:** 10.1186/s12870-020-02761-1

**Published:** 2021-01-27

**Authors:** Chunji Jiang, Xinlin Li, Jixiang Zou, Jingyao Ren, Chunyi Jin, He Zhang, Haiqiu Yu, Hua Jin

**Affiliations:** 1 College of Agriculture, Shenyang Agricultural University, Shenyang, 110000 China; 2 College of Environment and Resources, Dalian Minzu University, Dalian, 116600 China

**Keywords:** RNA-seq, Phytohormone, peanut *Arachis hypogaea* L., Drought stress

## Abstract

**Background:**

The peanut is one of the most important oil crops worldwide. Qualities and yields of peanut can be dramatically diminished by abiotic stresses particularly by drought. Therefore, it would be beneficial to gain a comprehensive understanding on peanut drought-responsive transcriptional regulatory activities, and hopefully to extract critical drought-tolerance-related molecular mechanism from it.

**Results:**

In this study, two peanut *Arachis hypogaea* L. varieties, NH5 (tolerant) and FH18 (sensitive), which show significantly differential drought tolerance, were screened from 23 main commercial peanut cultivars and used for physiological characterization and transcriptomic analysis. NH5 leaves showed higher water and GSH contents, faster stomatal closure, and lower relative conductivity (REC) than FH18. Under the time-course of drought-treatments 0 h (CK), 4 h (DT1), 8 h (DT2) and 24 h (DT3), the number of down-regulated differential expressed genes (DEGs) increased with the progression of treatments indicating repressive impacts on transcriptomes by drought in both peanut varieties.

**Conclusions:**

Nevertheless, NH5 maintained more stable transcriptomic dynamics than FH18. Furthermore, annotations of identified DEGs implicate signal transduction, the elimination of reactive oxygen species, and the maintenance of cell osmotic potential which are key drought-tolerance-related pathways. Finally, evidences from the examination of ABA and SA components suggested that the fast stomatal closure in NH5 was likely mediated through SA rather than ABA signaling. In all, these results have provided us a comprehensive overview of peanut drought-responsive transcriptomic changes, which could serve as solid foundation for further identification of the molecular drought-tolerance mechanism in peanut and other oil crops.

**Supplementary Information:**

The online version contains supplementary material available at 10.1186/s12870-020-02761-1.

## Background

The peanut (*Arachis hypogaea* L.) is one of the main sources of oil and protein in the diet of humans. Its rich nutritional value is especially beneficial to the human cardiovascular system. Peanut plantation are distributed widely across developing countries from semi-arid tropical to subtropical regions [1, 2]. Historically, peanuts have played important roles in the Chinese agricultural economy and are still the current top ranking Chinese exported crops. The annual Chinese peanut output had reached 1.3 × 7^10^ tons in 2008 [3]. Nevertheless, peanut quality and yield are often seriously diminished by drought. Annual worldwide losses in peanut production caused by drought is approximately six million tons [4]. The global drought which is on the rise today has exhibited a tendency of higher frequencies, longer duration and wider ranges. Also the frequency and severity of global drought are projected to keep progressing to severer levels in the next 30–90 years [3].

Once struck by drought, the normal growth progress of crops will be prohibited leading to yield reduction and even no-grain harvest. Up to date, studies have shown that drought stresses affect various biological processes, including water physiology, nutrient absorption, enzyme activity, photosynthesis and assimilate transport [5–7]. Plants under drought stresses can adjust their morphological, physiological and metabolic processes by changing gene expression patterns [8]. Generally, the expression of certain transcription factors (TFs) can be regulated by plant hormonal signals, then multiple stress-responsive genes are induced [9–11]. More specifically, drought stresses usually stimulate abscisic acid (ABA), ethylene (ETH) and salicylic acid (SA) signaling pathways which can direct plants to produce osmo-regulatory substances to maintain cell osmotic potentials and antioxidant enzymes to re-establish the oxidation balances [12–14]. In addition, plants can also close stomata, thicken cuticles, and harden cell walls to increase drought tolerance.

Until recently, transcriptomic studies have been conducted to gain insights into the molecular mechanisms underlying various perspectives of peanut biology. For example, Chen et al. have sequenced transcriptomes in young pods of peanut variety Yueyo7 trying to study why the development of young pods can initiate only when they reach the soil [15]. Also, Wu et al. have used leaves, stems and roots from the Spanish peanut *A. hypogaea* L to characterize peanut different developmental stages by transcriptomic analysis [16]. In addition, Cui et al. have sequenced transcriptomes in salt-stressed LH14 shoot and root tissues to investigate impacts of salt-stress on peanuts [17]. In comparison, there are only a few transcriptomic studies reported which aim at drought-related molecular mechanisms in peanuts. Shen et al. have studied drought-stressed transcriptomes in leaves of FH1 a drought-tolerant variety, which revealed transcriptional changes after seven-day drought treatments [18]. Another study by Brasileiro et al. havs analyzed transcriptomes from wild-peanut tissues which were stressed for eleven-days [19]. On the other hand, Zhao et al. have specifically studied peanut transcriptomic responses to shorter-drought (two-days) in root tissues from J1, the other characterized drought-tolerant peanut variety [20]. Taking above described three drought-transcriptomic studies into consideration, evidences have demonstrated that drought stresses could induce the differential expression changes of a suite of genes such as ABA-related, carbon metabolism-related, proline-related and photosynthesis-related genes. Nevertheless, molecular researches on drought-tolerance mechanisms in peanut is still in a preliminary stage especially because of its huge allotetraploid genome size.

Transcriptome sequencing technology has become an important tool for analyzing the molecular mechanisms of drought tolerance in plants. At present, RNA-Sequencing (RNA-Seq) can provide rich information on DEGs, transcript structures, new transcripts and isomers, alternative splicing and allele-specific expression etc. [21]. RNA-Seq has been successfully applied to analyze drought-tolerance molecular mechanisms in cuckoo, Yerba Mate and cotton [8, 22, 23], as well as other crop plants such as lentils, buckwheat and millet [24–26]. These studies have enriched us with great amount of helpful information on plant tolerance to drought stresses at the transcriptional level.

Transcriptomic comparison between varieties with significantly different stress tolerance is proved to be an effective strategy for analyzing molecular stress-responses in a certain crop [27]. Since early rather than late drought-responses usually indicate the up-stream regulatory events within the whole drought-responsive mechanism, it would be especially valuable to fill the blank in knowledge on early drought-induced peanut molecular dynamic changes. Therefore, we chose two commercial peanut varieties that demonstrated differential drought tolerance in our screening as study materials (FH18, the sensitive type, and NH5, the tolerant type). PEG-6000 treatments during the seedling stage were adopted to simulate drought stress conditions. Physiological indexes were further measured to monitor the physiological status of peanut seedlings under continuous drought stress. The RNA-Seq technology was applied to analyze leaf transcriptomes of FH18 and NH5 at different stress time-points. The peanut transcriptomic spectrum under drought stress was studied, from which insights into the molecular mechanism of peanut drought tolerance in the seedling stage were expected to be gained.

## Results

### Peanut drought-tolerance

**Fig. 1:**
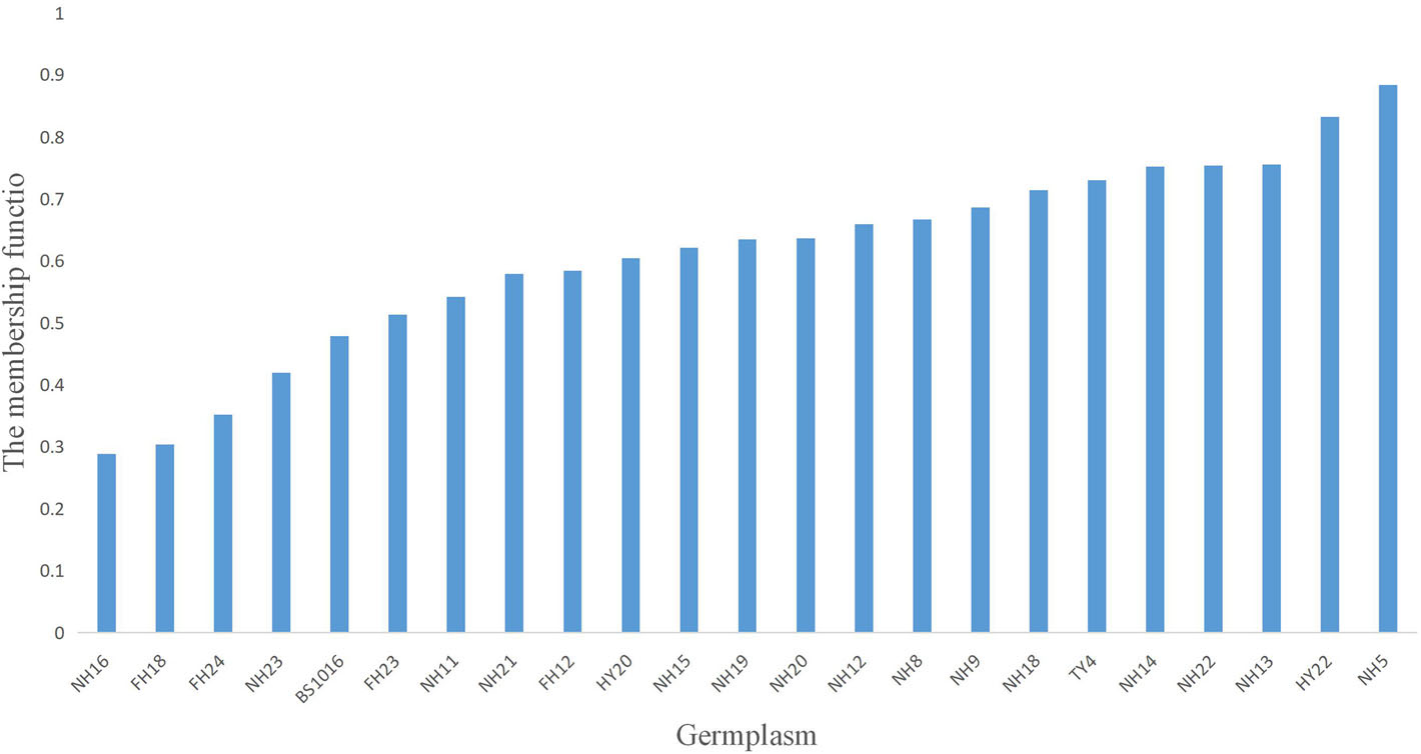
Comprehensive evaluation of drought tolerance of peanut under drought stress

In recent years, the plantation acreage of peanuts in the northeastern provinces of China has constantly increased. To evaluate the performance of the current peanut germplasms in drought conditions and to search for suitable research materials for the study of peanut drought biology, we examined 23 representative commercial peanut varieties for their drought tolerance. After 24 h of simulated drought stress, all tested3 varieties exhibited differenced in relative fresh weight (FW), wilting index (WI), leaf water loss, and conductivity (Table S1). The level of drought-tolerance was represented by a calculated “membership function” (as described in the “materials and methods”). Using this approach, the most drought-tolerant varieties were NH5 and HY22, with ratings of 0.884 and 0.833, respectively. The least drought-tolerant varieties were FH18 and NH16 with ratings of 0.304 and 0.288, ~ 36% of NH5 (Fig. 1). Therefore, FH18 and NH5 were chosen as drought-sensitive and drought-tolerant peanut varieties for further analysis also because their development paces synchronized with each other.

### Analysis of peanut drought-responses

**Fig. 2:**
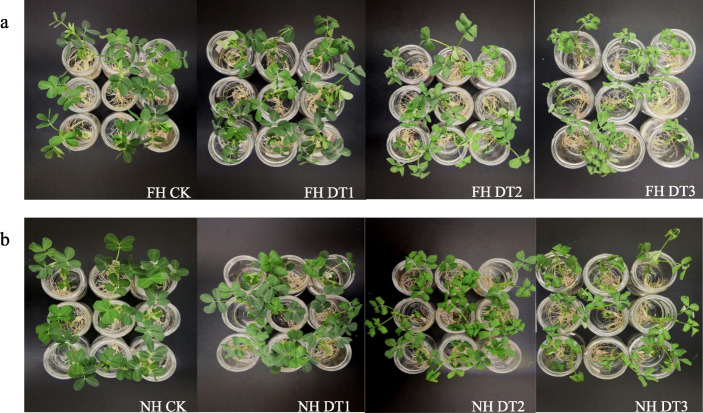
Phenotypic analysis of drought-stressed FH18 and NH5 seedlings. **a** FH18 seedlings at 0 h, 4 h, 8 h, 24 h drought-treatments; (**b**) NH5 seedlings at 0 h, 4 h, 8 h, 24 h drought-treatments

Since both FH18 (sensitive type) and NH5 (tolerant type) seedlings showed vigorous growth during the 4th-leaf stage (Fig. 2), seedlings at this stage were examined for phenotypic changes caused by continuous simulated drought-stresses. First, leaves from both varieties had exhibited obvious wilting when the drought treatment prolonged. However, FH18 leaves wilted to a severer extent than those of NH5 (Fig. 2). For example, FH18 leaves started drooping at DT1 (4 h), while no obvious change could be observed in NH5 leaves at the same time. At DT2 (8 h), FH18 leaves significantly wilted but NH5 leaves only partially wilted (Fig. 2). These observations indicated that NH5 could preserve higher leaf water-contents under drought conditions than FH18.

**Fig. 3:**
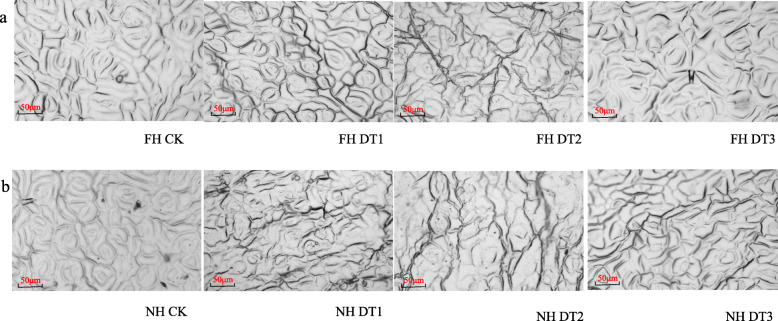
Stomatal analysis of drought-stressed FH18 and NH5 leaves. **a** Stomata in FH18 seedlings under 0 h, 4 h, 8 h and 24 h drought-treatments; (**b**) Stomata in NH5 seedlings under 0 h, 4 h, 8 h and 24 h drought-treatments

Stomata are vital gateways for plants to control carbon and water exchange between the leaf surface and the atmosphere. Based on the above observations, it was expected that different stomatal closure patterns would be identified between FH18 and NH5 during the different drought treatment time-periods. As expected, the stomata of both peanut varieties remained open at 0 h of drought stress (Fig. 3). NH5, but not FH18, showed stomatal closure at DT1 (Fig. 3). At DT2 and DT3, the stomata in both peanut varieties were all closed (Fig. 3). These results suggest that drought conditions induced a quick stomatal closure in NH5 leaves but not in the FH18 leaves, which may have contributed to the observed slower water loss and higher leaf water content in NH5 compared to FH18 (Fig. 2).

**Fig. 4:**
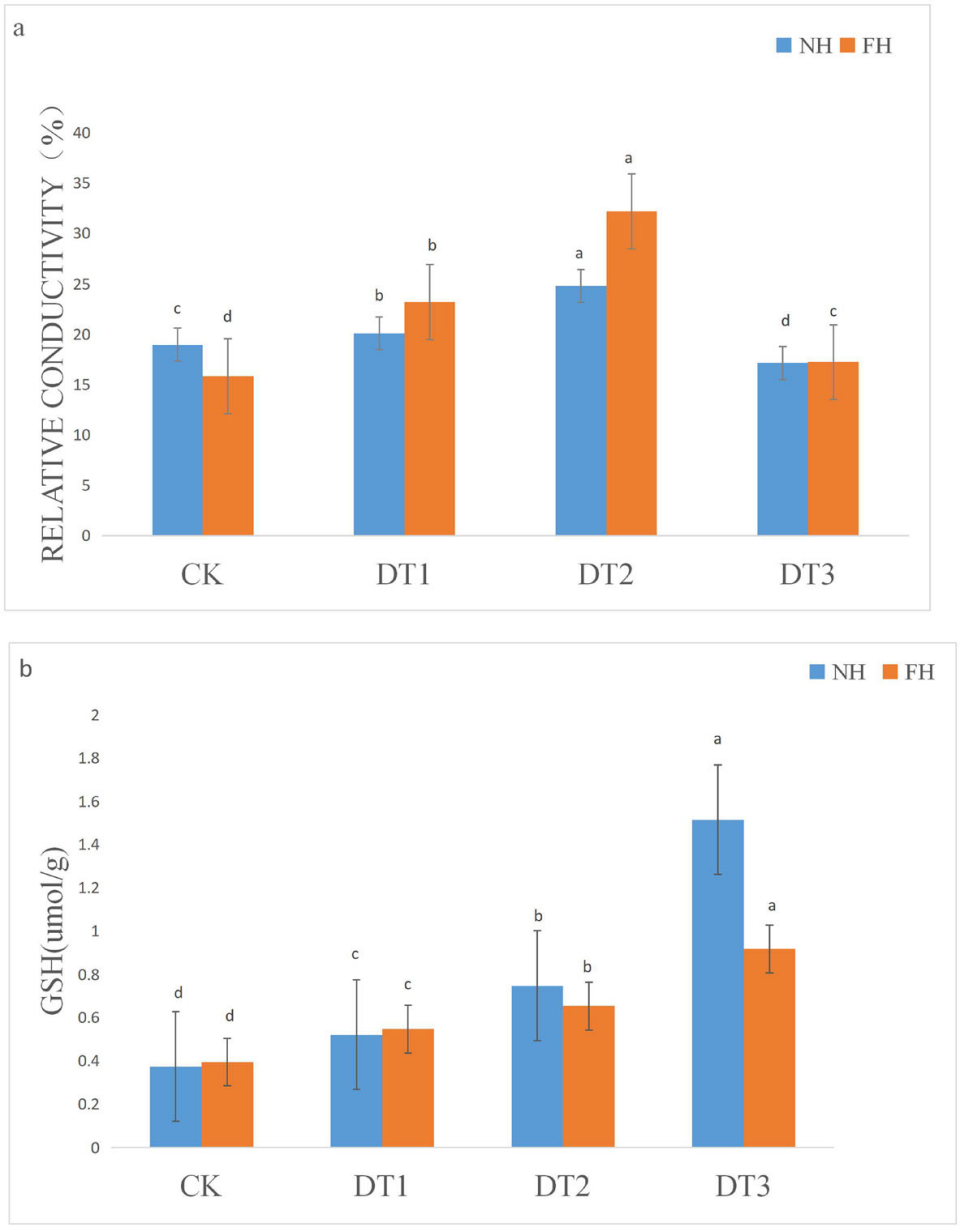
Determination of physiological indexes of FH18 and NH5. **a** Conductivities in FH18 and NH5 leaves; (**b**) GSH contents in FH18 and NH5 leaves

Relative conductivity (REC) is an index which is used to reflect the osmotic-adjustment in the plasma membrane to stresses. Under drought conditions, a lower REC value correlates with an increased ability to adjust the osmotic balance. This allows for a higher drought tolerance. As shown in Fig. 4a, the REC values of NH5 were lower than those of FH18 at DT1 and DT2 (relative REC increased compared with CK: 1.81% in NH5 and 7.36% in FH18 at DT1; 5.85% in NH5 and 16.36% in FH18 at DT2) (*P* < 0.01). These data suggested that NH5 preserved better plasma membrane osmotic adjustment ability than FH18.

Reduced glutathione (GSH) is one of the most effective scavengers for reactive oxygen species (ROS). The GSH contents in FH18 and NH5 samples were determined (Fig. 4b). Under control conditions, there was no significant difference in GSH content between the two peanut varieties. As the drought treatments progressed, the GSH content increased in both peanut varieties but the magnitude of these increases differed. Compared with the CK group, the GSH content in NH5 at DT1, DT2, and DT3 increased by 0.15 mol/g, 0.37 mol/g and 1.4 mol/g respectively, while in FH18 at DT1, DT2, and DT3 it increased by 0.15 mol/g, 0.26 mol/g and 0.52 mol/g, respectively (*P* < 0.01), about 40% of that of NH5 at DT3. These results showed that drought stresses induced higher GSH contents in NH5 and therefore NH5 contained stronger ROS scavenging capabilities than FH18.

### Transcriptome sequencing and assembly

Transcriptomes from the FH18 and NH5 seedlings, which underwent different levels of stress, were sequenced using Illumina 2000, and a total of 24 transcriptome libraries were constructed (three library repeats for each variety at every time-point). After removing the low-mass readings, 177.69 Gb of clean data were obtained. The clean data for each sample reached 5.90 Gb and the percentage of Q30 bases was 94.62% or more. The clean reads for each sample were aligned with the designated reference genome, and the alignment efficiency ranged from 94.47 to 97.49%. Based on comparisons, alternative splicing prediction analysis, and gene structure optimization analysis, 6940 new genes were discovered (Table S2).

### Expression analysis

**Fig. 5:**
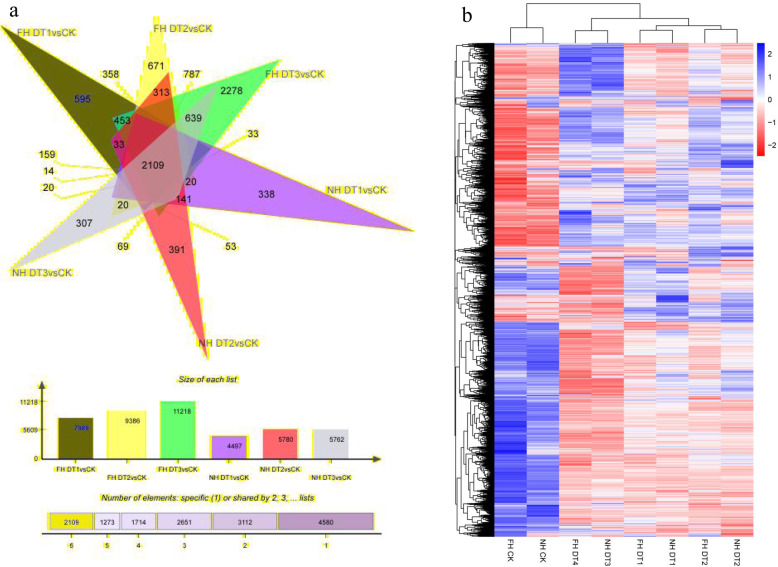
Differentially expressed genes between FH18 and NH5 under drought stress. **a** Venn map of differentially expressed genes in two species of peanut under drought stress, and (**b**) thermographic analysis of transcriptional levels of differentially expressed genes in two species of peanut under drought stress

Drought stresses can induce significant changes in gene expression patterns. Therefore, differentially expressed genes (DEGs) among our sequenced samples were extracted according to their differential expression levels. Then, functional annotation and enrichment analysis were carried out with these identified DEGs. DEGs for FH18 at DT1, DT2 and DT3 were respectively identified as 7989 (up-regulated 3709/down-regulated 4280), 9386 (up-regulated 4052/down-regulated 5334) and 11,218 (up-regulated 4881/down-regulated 6337). In contrast, DEGs for NH5 were 4497 (up-regulated 2448/down-regulated 2049) at DT1, 5780 (up-regulated 2673/down-regulated 3107) at DT2 and 5762 (up-regulated 2585/down-regulated 3177) at DT3. It was obvious that at each time point DEGs for FH18 significantly out-numbered those for NH5. For example, the number of FH18 DEGs at DT3 was 11,218 almost twice of NH5 DEGs. These DEGs-number differences illustrated that drought stresses could induce more dynamic transcriptomic changes in the FH18 genome than in the NH5 genome. In another word, NH5 seemed to be able to maintain more stable transcriptomes under drought conditions. Furthermore, the number of down-regulated FH18 DEGs was ~ 30% more than the number of up-regulated DEGs at both DT2 and DT3. As of NH5 DEGs, these ratios were ~ 15% at DT2 and ~ 20% at DT3. These results suggested that drought- stresses within 24 h exerted more down-regulatory impacts on peanut transcriptomes. In addition, this drought-induced down-regulatory impact on transcriptomes appeared to be relatively minor for NH5 than for FH18. Taken together, the differences in DEGs between NH5 and FH18 provided a justified reflection of different molecular basis underlying NH5 drought-tolerant and FH18 drought-sensitive phenotypes. Last, cluster analysis was carried out with identified differential genes (Fig. 5b).

### Functional annotation of DEGs

**Fig. 6:**
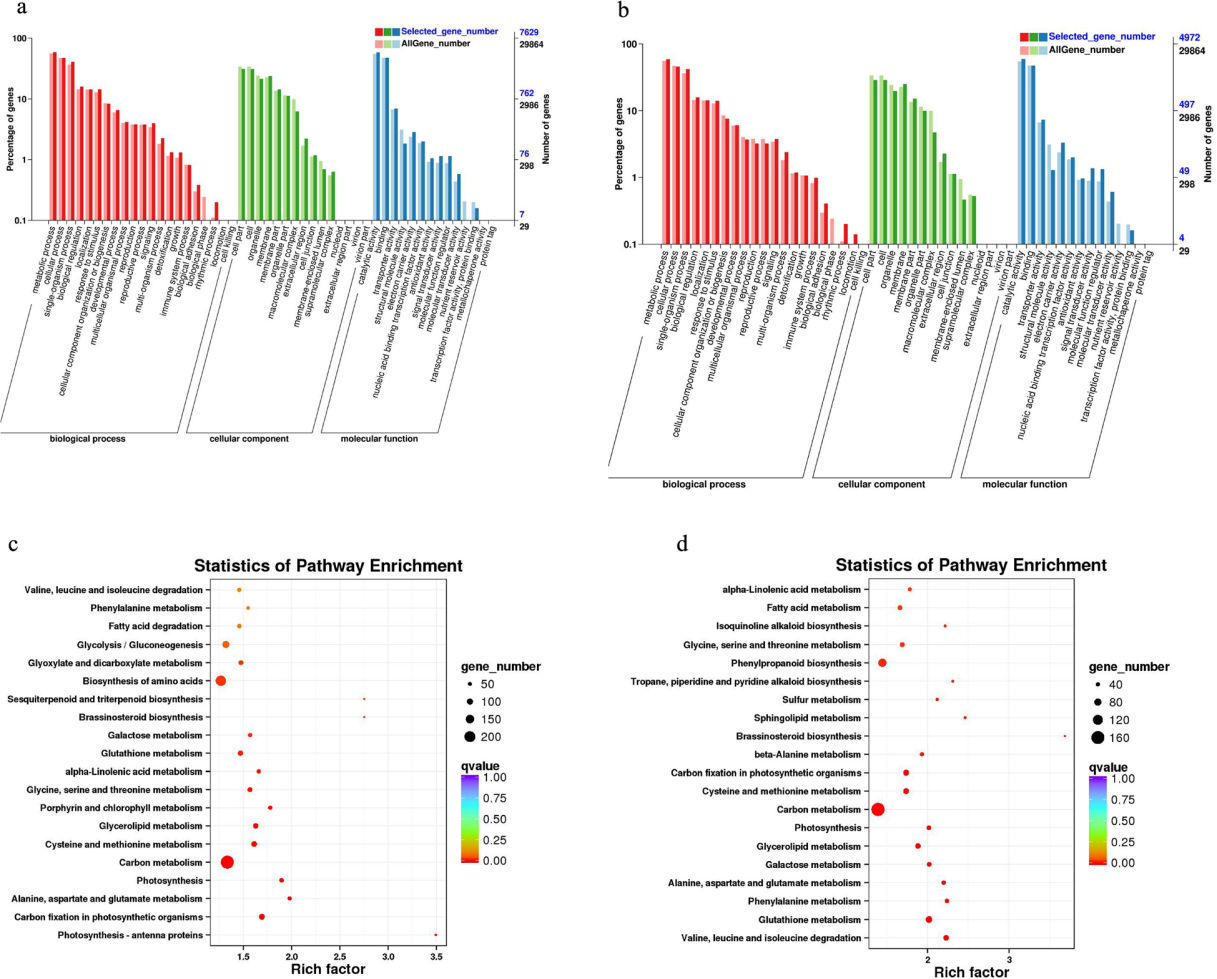
Functional annotations of DEGs in drought-stressed FH18 and NH5 leaves. **a** GO classification of DEGs in FH18; (**b**) GO classification of DEGs in NH5; (**c**) KEGG pathway enrichment and dispersion map in FH18; (**d**) KEGG pathway enrichment and dispersion point map in NH5

Functional annotation was carried out for identified DEGs (refer to Table S3 for statistical numbers of genes annotated in each differential gene set). GO classification was respectively applied to DEGs in FH18 and NH5. The matched DEGs were divided into three functional categories: biological processes, molecular functions, and cell components (Fig. 6a and b). In the category of biological processes, the most abundant genes belonged to “metabolic processes” and “cellular processes”. In the category of cell components, the number of genes in “cell parts and cells” was the highest. In the category of molecular function, DEGs mainly belonged to “binding” and “catalytic activity” subgroups. In order to identify active biological pathways enriched with DEGs in both peanut varieties, the KEGG pathway database was searched (Fig. S1). The results of the KEGG enrichment analysis are shown in Fig. 6c and d with the first 20 top-ranking pathways indicated by the smallest significant Q values. Although FH18 and NH5 shared similar pathway enrichment patterns, the number of enriched genes and the expression levels of enriched genes were quite different (Table S4 and S5). The enriched pathways included GSH-related glutathione metabolism, glycolysis, glyoxylic acid, and dicarboxylic acid ester metabolism associated with pyruvic acid. Pathways of corneal and wax anabolism; fatty acid degradation related to the stratum corneum; carbon fixation; photosynthesis-antenna protein; photosynthesis; degradation of valine, leucine, and isoleucine amino acids; and the porphyrin and chlorophyll metabolisms were also enriched. In addition, several pathways were only enriched in the drought-tolerant variety, NH5: alanine metabolism; sulfur metabolism; sphingolipid metabolism; phenylpropane biosynthesis; isoquinoline alkaloid biosynthesis; and the biosynthesis of tropane, piperidine, and a pyridine alkaloid.

### Peanut drought tolerance-related genes and pathways

**Fig. 7:**
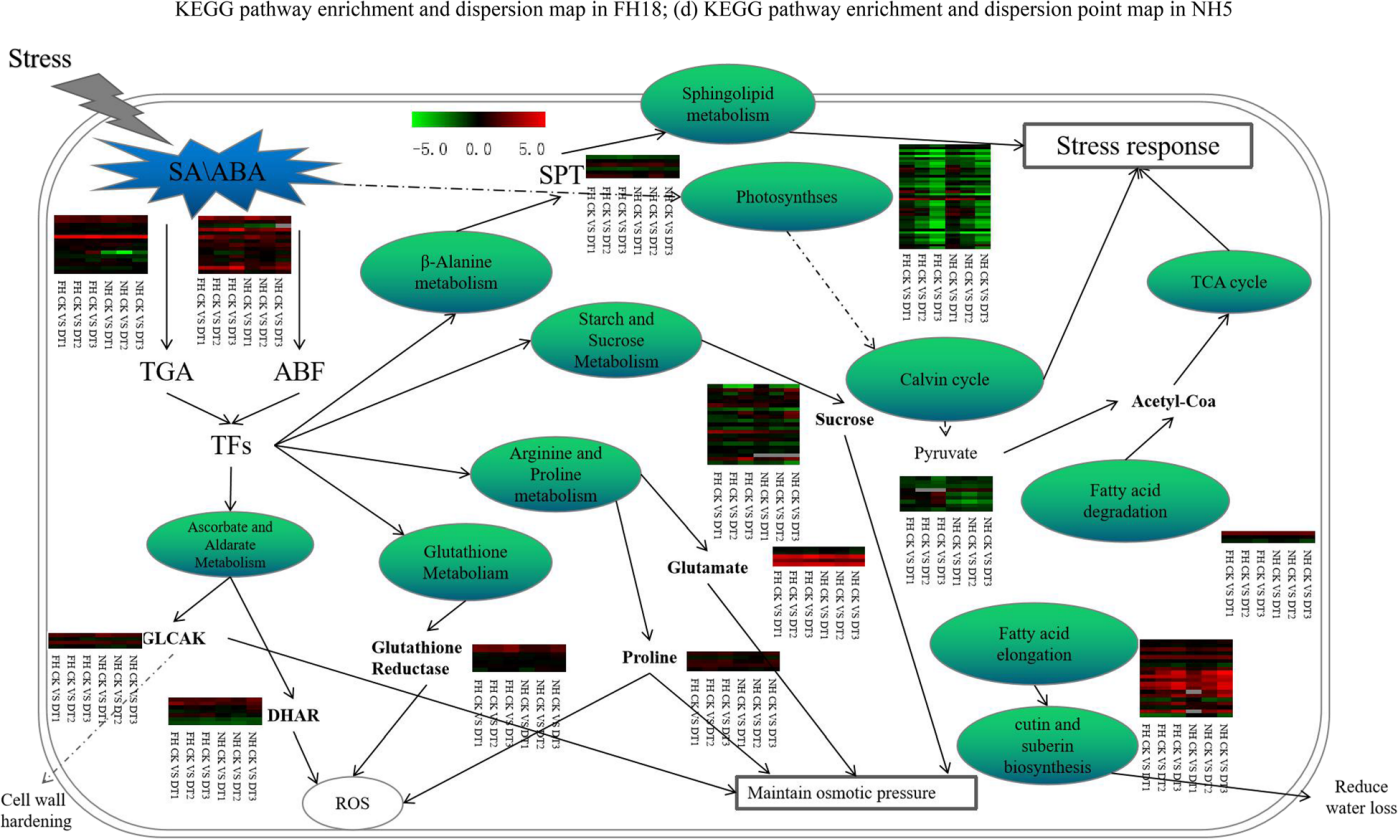
The schematic diagram of main peanut drought-responsive processes. It showed that peanut can resist drought stress by regulating the expression of stress genes through ABA and SA signaling. Thermography showed that FH18 and NH5 responsive genes were up-regulated (red) and down-regulated (green) under drought stresses

In order to explore the drought-tolerance mechanism in peanut, we examined transcriptional changes of potential drought-tolerance genes in FH18 and NH5. We found that genes related to ABA and SA signal-transduction were significantly up-regulated, including sixteen ABF genes and twenty-two TGA (TGACG motif-binding factor) genes (Table S6). Compared with FH18 transcriptomes, some genes were differentially expressed only in NH5. These NH5-specific DEGs could be categorized into various biological pathways. Among them, fourteen genes were identified as ROS-scavenging genes (Table S7) belonging to glutathione metabolism and proline metabolism. Thirty-three osmotic-potential-regulating genes (Table S7) were under the metabolism of arginine, proline, sucrose and starch. Fourteen cell wall sclerosis-related genes and fourteen cutin and wax metabolism genes were also enriched from NH5 transcriptomes which were suspected to affect water loss (Table S7). Another set of genes involved in peanut defense-responses showed much higher expression levels in NH5 than in FH18. On the other hand, FH18-specific differential genes were also identified. However their expression patterns indicated that these genes were suppressed by drought treatments. Furthermore, another 126 DEGs were found to be enriched in the main drought-responsive metabolic pathways (Table S7) such as the sphingolipid metabolism, photosynthesis, the pyruvate metabolism, fatty acid degradation, and the tricarboxylic acid cycle. A diagram of the interactions of the above-described enriched-pathways is shown in Fig. 7.

### Real-time qPCR validation

**Fig. 8:**
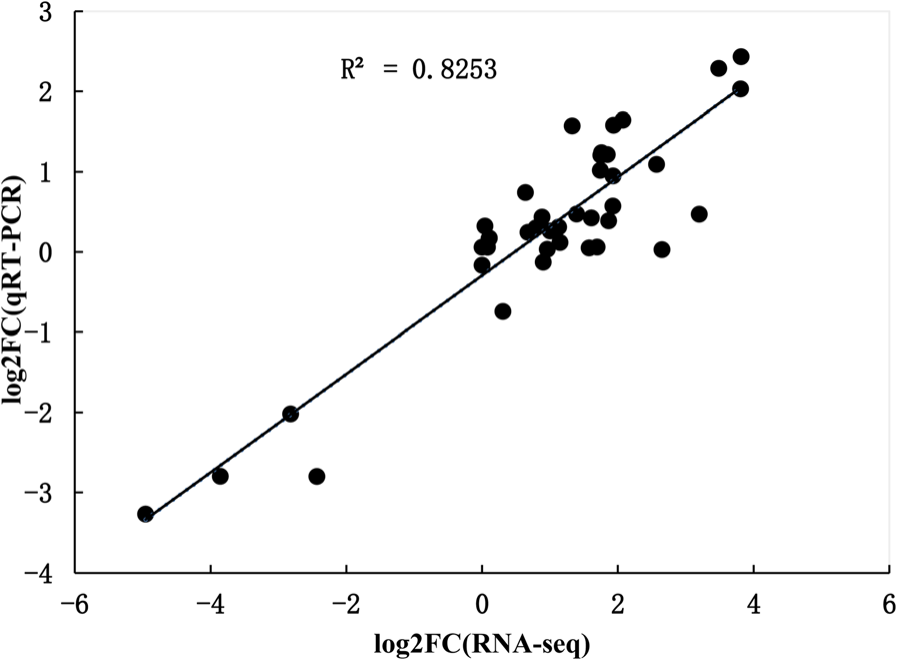
Correlation between RNA-Seq expression profile and qRT-PCR results

In order to validate the transcriptome data sets, the real-time qPCR technology was applied to analyze transcriptional levels of ten genes which were randomly selected from drought-tolerance-related pathways. The relative expression levels of genes were measured and calculated using *ARAH1* as the internal reference gene. These ten genes included: *pyruvate dehydrogenase*; *glutamate synthetase*, *agmatine deiminase isoenzyme X2*, *PXG*, *trehalose 6-phosphate synthase/phosphatase*, *inositol oxygenase 2*, *glutathione S-transferase*, c*innamyl alcohol dehydrogenase*, *glycerol kinase* and *enoyl-CoA hydratase*. The RT-PCR results confirmed that the transcription changes of these 10 genes were comparable with the fold-changes observed in our transcriptome analysis (Fig. 8).

## Discussion

### Adaptation of peanuts to drought stress

Drought stress is one of the main limiting factors for crop growth and productivity. In general, plant drought-tolerance involves the combination of a variety of physiological and biochemical changes which are based on the coordinated expression of a hierarchy of genes. This complex mechanism is the result of interaction between plant heredity and the external environmental changes [28–30]. In this study, we used PEG-6000 to simulate drought stresses in combination with the transcriptome sequencing technology to analyze the drought-tolerance in two peanut varieties (FH18 and NH5). Compared to FH18, the drought-tolerant variety NH5 showed stronger capabilities of adjusting osmotic-potential in the plasma membrane and scavenging reactive oxygen species (ROS). We also observed drought-induced stomatal closure to reduce water loss in FH18 and NH5, particularly the quicker stomatal closure in NH5 at 4 h drought-treatment.

### Stratum corneum biosynthesis and cell-wall sclerosis

The stratum corneum is a membrane structure which can protect plants in stressful environment. The biosynthesis of stratum corneum determines its properties such as water permeability, and therefore it is an efficient water-saving mechanism for plants to control water-loss through affecting the stratum corneum composition [31–33]. The stratum corneum is composed of waxes, cutin and polysaccharides. Waxes consist of various aliphatic molecules, mainly long-chain fatty acids (VLCFAs) containing more than twenty carbon atoms and their derivatives include primary alcohols, secondary alcohols, aldehydes, alkanes, ketones and wax esters [34]. Consistent to findings in bread wheat [35], we also found that wax-biosynthesis-related genes were able to be induced by drought. For instance, fatty acyl-CoA reductase (FAR) was a highly expressed DEG identified in our study. The biological function of FAR was proposed to be supplying primary alcohol for wax-biosynthesis since previous research had shown that wheat lines lacking *TaFAR1* contained significantly reduced levels of primary alcohol in its leaf blade and anther wax [36]. Typical cutin is represented by epoxy C16/C18 fatty acids, which are cross-linked by ester bonds to form elastic polyester structures [37, 38]. In this study, we found that the transcriptional abundances of C18/C22 synthetic genes were significantly induced by drought stresses. It has been speculated that the drought-stressed peanut stratum corneum may be mainly composed of C18 fatty-acid cutin and docosan-acid wax [37]. Our results provided strong evidences which suggested that the biosynthesis of stratum corneum was an important drought-tolerance mechanism. In addition, we had found that the drought- induction of cutin- and wax-related genes was stronger but slower in NH5 than in FH18. Taking the better drought-tolerance of NH5 into consideration, we were confident to propose that the stratum corneum played its water-saving function mainly under prolonged drought conditions.

Cell-wall hardening in leaves is another known main drought–response for plant. Drought-stressed plants usually contain lower water-potentials and exhibit higher levels of cell-wall hardening. The hardening of plant cell-walls can effectively lead to reduction in leaf growth and in water transpiration. Covalently combined lignin and hemicellulose molecules can form interwoven networks which is the molecular basis for plant cell-wall hardening. It was reported that both soybean and Triticale accumulated lignin to harden cell-walls under drought conditions [39, 40]. The biosynthesis of lignin can be affected by drought stresses through the regulation of the phenylpropane biosynthesis pathway. The induced phenylpropane biosynthesis is also able to affect the biosynthesis of anthocyanins which in turn will promote the formation of plant keratins [41]. In this study, we found that phenylpropane biosynthetic pathway was only enriched in NH5 but not in FH18 samples. Therefore, the phenylpropane biosynthesis might be a significant molecular mechanismfacto underlying why NH5 is more drought-tolerant than FH18.Glucosyluronic acid kinase (GLCAK) is a gene which participates in the precursor-synthesis of pectin and hemicellulose [42]. According to Xiao et al. the arabidopsis GLCAK mutant (deletion mutant) has exhibited lower drought-tolerance and soluble-sugar content than WT [42]. In this study, the drought-induction of GLCAK was observed. This GLCAK induction might be able to lead to the cell-wall hardening and the accumulation of soluble sugars in peanut cells which could balance osmotic-potentials and help to resist drought stresses.

### Steady osmotic-potential and ROS scavenging

The regulation of plant osmotic-potentials is a defensive mechanism against drought stresses [43]. Under drought conditions, osmotic-adjusting substances will accumulate in plants, maintaining the balance of cellular osmotic-potential, turgor pressure and cell volume [44]. Proline is a protective osmotic- regulatory agent. High levels of proline can reduce the water potential and enhance the ROS removal by antioxidants as in peas and Stipa purpurea [45, 46]. Sucrose, a soluble sugar, also plays an important role in the plant osmotic-regulation. The accumulation of soluble sugars can enhance the water absorption into cells [40, 47]. Similar to the finding of the present study, chieh-qua can enhance its drought tolerance by enhancing carbohydrate metabolism gene expression [48]. Glutamine can also function as another osmotic-regulator in resisting drought stresses [49]. The results from our study had suggested that the induction of synthetic genes of proline, sucrose and glutamic acid might be the molecular basis for maintaining the balance of osmotic-potential in drought-stressed peanuts.

Plants tend to accumulate reactive oxygen species (ROS) under drought stresses. ROS can peroxidize plasma membrane leading to cell death in severe cases [50]. On the opposite, antioxidants are also often observed to accumulate in drought-stressed plants, such as MDA in millet [26] and flavonoids in barley [51]. Glutathione reductase (GR) and dehydroascorbate reductase (DHAR) are antioxidant enzymes which can effectively scavenge free radicals and protect plant organisms [52, 53]. GR can reduce oxidized-glutathione (GSSH) to reduced-glutathione (GSH) which is the scavenger for free radicals and particular organic peroxides [54, 55]. In our study, GR and DHAR genes were found to be up-regulated under drought conditions. Additionally, the transcription of GSH in the tolerant variety NH5 was higher than that in the sensitive variety FH18. All these findings suggested the vital involvements of glutathione and ascorbic acid in the peanut drought-tolerance mechanism.

### ABA and SA signal transduction pathways

Plants usually respond to external stimuli by activating signaling cascades which modify downstream gene expression patterns and finally realize physiological and metabolic adaptations [56]. Abscisic acid (ABA) and salicylic acid (SA) signaling pathways were found to be significantly induced by drought in this study. ABA and SA are two well-known plant hormones which play key roles in triggering drought-responses [57–59]. The core ABA signaling factors include ABA receptors (PYL/PYR), protein phosphatase 2C (PP2C), SNF1-related kinase (SNRK2) and ABA response-element-binding-factors (ABFs). Under drought conditions, ABA binds to PYLs/PYRs to inhibit PP2C which leads to the promotion of SnRK2. Then SnRK2 activates ABFs to regulate downstream transcription factors and to initiate ABA responses [34, 60]. Drought often induces an elevated ABA level in plant which will cause the binding of ABI1 (Abel son interactor protein 1) to PYL/PYR receptors. Once ABI1 binds to PYLs/PYRs, the inhibition of SLAC1 kinase by ABI1 will be released, which in turn will result in the closure of anion channels and eventually stomatal closure [61, 62]. For example, the ABA content of pearl millet increases under drought, which regulates the opening and closing of pores, reduces water loss and maintains the moisture content [63]. In the present study, the transcription of an ABA-biosynthesis-related gene NCED in both FH18 and NH5 varieties was found to be significantly induced by drought treatments indicating an elevation in the ABA level. On the other hand, the ABA-receptor PYL/PYR-related genes were repressed in both NH5 and FH18 by drought-treatments. Furthermore, our results showed that the negative ABA signaling regulator PP2C was also induced and the positive component SNRK2 was repressed by drought treatments, suggesting a decrease in ABA-sensitivities. Nevertheless, the transcriptional levels of ABFs, ABA-down-stream transcription factors, were significantly induced in this study. These seemingly confusing results on the whole ABA-signaling cascade were exactly the evidences for acknowledging the complicate and intricate involvement of ABA-signaling in peanut drought-tolerance mechanisms. Lastly, the drought-repression on PYL/PYR-related genes suggested that the PYL/PYR–mediated SLAC1 release would be repressed. Therefore, SLAC1-mediated stomatal-closure should also be repressed. Since both NH5 and FH18 varieties showed stomatal-closure responses especially the quick closure in NH5, it was reasonable to postulate that this stomatal-closure response might not be mediated through the PYL/PYR molecular module.

Studies have shown that SA application to barley plants can enhance their drought-tolerance [64]. Several other reports have also demonstrated the protective effects of exogenous application or endogenous accumulation of SA against drought stresses [65]. The SA is biosynthezed in peanut through the phenylalanine pathway of which the rate-limiting reaction is catalyzed by phenylalanine ammonia lyase (PAL). Previous research has shown that drought stresses can increase the SA contents by increasing PAL activity, thus improving plant drought-tolerance [66]. TGA family transcription factors are downstream components of the salicylic acid (SA) pathway and play an important role in plant water stress defense [67]. Also as Miura et al. have pointed out that SA can promote stomatal-closure and induce the expression of defense-genes [58]. In this study, PAL and TGA genes were highly expressed indicating that the SA signaling participated in peanut drought-responses. Although SA might dominate peanut stomatal closure, some TGA genes were only induced in NH5, which may explain why NH5 stomatal closure was faster than FH18. All these findings on the drought-induction of ABA- and SA-related genes strongly implicated that the hormonal signaling in drought-stressed peanuts was initiated by both ABA and SA hormones, thus comprised a highly complex drought-combating molecular mechanism in peanuts.

## Conclusion

In conclusion, we first characterized the physiological responses of drought stressed peanuts. We then obtained peanut transcriptome data sets of different genetic materials using RNA-Seq technology, in order to explore the key drought-related genes and metabolic pathways. Our results showed that the ABA- and SA-signaling were activated in peanuts under simulated drought stress. The expression patterns of genes related to stratum corneum biosynthesis, cell wall hardening, ROS clearance and osmotic-potential were also changed in favor of resistance to drought stress. All these findings expanded our knowledge of peanut drought-tolerance mechanisms and could facilitate future breeding of elite peanut germplasms.

## Methods

### Materials and growth

Twenty-three major commercial peanut varieties in the Northeastern China were obtained from Shenyang Agricultural University. Sixteen of these have been formally identified by the national and local approval committee, respectively, and the others are under review. More detailed information is listed in Table S8. Peanut seeds were presoaked in deionized water and germinated in the dark for 24 h in a 28 °C incubator. Germinated seeds were planted in sand and grown under a 16 h/8 h light cycle, 60% humidity, and 28 °C supplemented with ½ Hoagland solution every other day. Seedlings at the 4th true-leaf stage with similar height were washed, dried and then root-cultured in Hoagland solution for another 3 days. The addition of 20% PEG-6000 to the Hoagland solution was adopted as the simulated drought condition and the untreated Hoagland solution was used as the control condition.

### Drought-tolerance screening

After 24 h treatment, stressed (S) and control (CK) seedlings were collected for the following measurements. All measurements were performed with three independent biological replicates, unless otherwise specified.

Determination of water-loss rate (RWL): The second compound leaf (1.0 g) was detached from plants and weighed immediately for FW_1_. Then, detached leaves were placed in the yarn net and air-dried for 2 h (kept from the wind and direct sunlight). Next, the air-dried leaves were weighed for FW_2_. The leaves were then dried in an oven at 80 °C to a constant weight (DW). The oven-drying time duration is represented as (t1 - t2). RWL was calculated using the following equation: RWL (mg·g^− 1^·min^− 1^) = (FW_1_-FW_2_) / DW(t_1_-t_2_).

Determination of relative plant fresh weight (RFW): firstly, the average fresh weights of seedling of drought-stressed and CK groups were respectively measured and calculated using three randomly chosen seedlings as independent biological replicates for each group. Relative plant fresh weight RFW was calculated as the follows: RFW = average fresh-weight of drought-treated plants / average fresh-weight of CK plants. Conductivity: The conductivity was measured using a conductivity meter (Orion-METTLER-FE30K) at room temperature (24 °C) and calculated as described by Xu et al. [30].

Determination of wilt index: the peanut wilt grades were visually evaluated. Peanut seeds were germinated as described above. Germinated seeds were planted in 15 cm-diameter flowerpots with the same amount of sand and under a 16 h/8 h light cycle, 60% humidity, and a temperature of 28 °C. Seedlings were supplemented with ½ Hoagland solution every second day. Once the third true-leaf stage was reached, watering was stopped, and the soil was allowed to dry naturally. When the soil reached 75% relative water content, digital pictures of peanut plants were taken every day. Namely, at grade 0: the peanut leaves were naturally expended and were bright and glossy; the culm was firm also. At grade 1: the leaves began to lose water, the leaves were dull, and the top one or two leaves were slightly drooping. At grade 2: the plants continued to lose more water, and the drooping of leaves increased. At grade 3: some leaves were dry, hard, and curly. At grade 4: all leaves were drooping and shrinking and had turned yellow. At grade 5: leaves were completely dry and hard, and the plants had died. If the wilting degree was between two levels, it would be treated as a grade and a half.

### Calculation of comprehensive index

The relative drought tolerance of peanuts was determined by the method of average “membership function” method [68].

The formula for “membership function” is as follows: μ_xj_ = (X_j_-X_min_)/(X_max_-X_min_), where, for a certain variety, μ_xj_ was the “membership function” for the “J” trait; x_j_ is the value of the “J” trait; and X_max_ and X_min_ are respectively the maximum and minimum values for the trait among all considered varieties. In order to avoid errors caused by variety differences, X_j_, X_max_ and X_min_ were all calculated as “relative values” instead of “measured values”. The relative value = the measured value under stress/ the measured value under the control.

### Drought-treatment time-course

FH18 and NH5 seedlings were prepared and treated as described in the “materials and growth” section. The drought-treatment time-period was composed of a series of treatment time points: 0 h (CK), 4 h (DT1), 8 h (DT2), and 24 h (DT3). The second compound leaves of seedlings were collected at each time point, which were frozen in liquid nitrogen and stored in a refrigerator at − 80 °C for further analysis.

### Physiological index measurements and stomatal observations

In order to examine drought-induced phenotype changes in FH18 and NH5, the second-compound leaf of seedlings were randomly selected from the treatment group and the control group, and then physiological indexes were measured. The conductivity was measured as described above. Reduced glutathione was measured by using a kit (Suzhou Keming Biotechnology Company) following the manufacturer’s protocols. Observation of peanut stomata was carried out on a Zeiss fluorescence positive microscope [69]. All measurements were performed using three independent biological replicates.

### RNA extraction and RNA-seq

RNA samples were prepared from 24 peanut leaf samples (4 treatments × 2 genotypes × 3 biological replicates). Total RNA was extracted using the TRIzol Reagent (Invitrogen, Carlsbad, CA, USA) following the manufacturer’s instructions. The quality of RNA was assessed by Agilent 2100. The method described by Wang et al. was adopted for sequencing [27]. Briefly, mRNA was fractionated and enriched using magnetic beads coupled with Oligo (dT). Single- and double-stranded cDNAs were synthesized from the mRNA using random hexamers and AMPure XP beads (Beckman Coulter, Beverly, CA, USA). PCR enrichment was then performed to obtain final cDNA libraries. In order to separate the cDNA fragments with a length of 240 bp, the library was purified by AMPure XP. The library quality was evaluated in Agilent Bioanalyzer 2100 system. Finally, Illumina 2000 was used to amplify the library.

### Data analysis

The built-in software “perl scripts(The perl scripts is provided by Biomarker Company (Beijing))” was used to clear the inferior quality readings from the original data. Before downstream analysis, the clean reads in each sample were mapped to the peanut reference genome database (https://www.peanutbase.org/data/public/Arachis_hypogaea/Tifrunner.gnm1.KYV3/arahy.Tifrunner.gnm1.KYV3.genome_main.fna.gz) using HISAT (version 2.0.4) software. All the genes were annotated using the NCBI non-redundant and Kyoto Encyclopedia of Genes and Genomes pathway (KEGG) databases. DESeq (version 1.10.1) was used for differential expression analysis and the clustered profiles with a *P*-value ≤0.01 and fold-change of ≥2 were considered as differentially expressed genes (DEGs) [70]. In order to analyze the functional relations of DEGs, we performed the GO (Gene Ontology) and KEGG enrichments based on GOseq R language pack and pathways in the KEGG database [71]. A hypergeometric test was used to test the enrichment-significance against the whole genomic background.

### QRT-PCR verification

To verify the accuracy of RNA-seq sequencing, ten putative drought-tolerance-related DEGs were randomly selected for qRT-PCR verification. The Arah1 gene was used as reference gene. Gene-specific primers were designed using PRIMER5(Table S9). QRT-PCRs were performed on an ABI Stepone plus platform. Each gene was analyzed in three biological samples, and three reaction repeats were performed for each biological sample.

## Supplementary Information

**Additional file 1: Fig. S1**. KEGG notes of differential genes between FH18 and NH5 under drought stress. (a)KEGG notes of differential genes in FH18 compared with CK; (b)KEGG notes of differential genes in NH5 compared with CK.

**Additional file 2: Table S1**. Phenotypic study of all tested varieties under PEG treatment.The table provides data on relative fresh weight (FW), wilt index (WI), leaf water loss and electrical conductivity of the tested varieties 24 h after simulated drought stress.

**Additional file 3: Table S2**. Summary of sequencing data quality. This table provides an overview of the sequencing data quality of peanut leaves under drought conditions.

**Additional file 4: Table S3**. Functional comments for DEGs. This table provides the result of functional annotation of the identified DEG.

**Additional file 5: Table S4**. Enrichment results by KEGG pathway in FH18. This table provides the number of enrichment genes and the expression level of enrichment genes.

**Additional file 6: Table S5**. Enrichment results by KEGG pathway in NH5. This table provides the number of enrichment genes and the expression level of enrichment genes.

**Additional file 7: Table S6**. Effects of drought on ABA and SA.This table provides expression levels of ABA and SA signal transduction related genes.

**Additional file 8: Table S7**. Drought-tolerance mechanism of peanut. This table provides expression levels of genes related to resistance to drought stress in peanut.

**Additional file 9: Table S8**. The peanut varieties used in the experiment. This table provides detailed information on the peanut varieties used in the experiment.

**Additional file 10: Table S9**. Primer sequences validated by qRT-PCR in this study. This table provides the primer sequences that were validated by qRT-PCR in this study.

## Data Availability

Raw data was deposited in NCBI database under SRA accession: PRJNA657965 (https://www.ncbi.nlm.nih.gov/sra/PRJNA657965).

## Abbreviations

cDNA: Complementary DNA
CK: Control group
DEGs: Differentially expressed genes
FPKM: Fragments per kb per million fragments
GO: Gene ontology
KEGG: Kyoto encyclopedia of genes and genomes
NCBI: National center for biotechnology information
qRT-PCR: Quantitative real-time PCR
RNA-seq: RNA sequence
ABA: Abscisic acid
SA: Salicylic acid
PAL: Phenylalanine ammonia lyase
PP2C: Protein phosphatase 2C
SNRK2: SNF1-related kinase
ABFs: ABA response element-binding factors
ABI1: Abel son interactor protein 1
GSSH: Oxidized glutathione
GSH: Reduced glutathione
GRG: Glutathione reductase
DHAR: Dehydroascorbate reductase
GLCAKG: Glucosyluronic acid kinase
ROS: Scavenging reactive oxygen species
