# Supplementary material for: Comparative transcriptome analysis of genes involved in the drought stress response of two peanut (Arachis hypogaea L.) varieties
Source: BMC Plant Biol. 2021 Jan 27;21:64. doi: 10.1186/s12870-020-02761-1 (PMC7839228; doi:10.1186/s12870-020-02761-1)
Supplement: Supplementary file 1 — Additional file 1: Fig. S1. KEGG notes of differential genes between FH18 and NH5 under drought stress. (a)KEGG notes of differential genes in FH18 compared with CK; (b)KEGG notes of differential genes in NH5 compared with CK. [file 12870_2020_2761_MOESM1_ESM.docx]

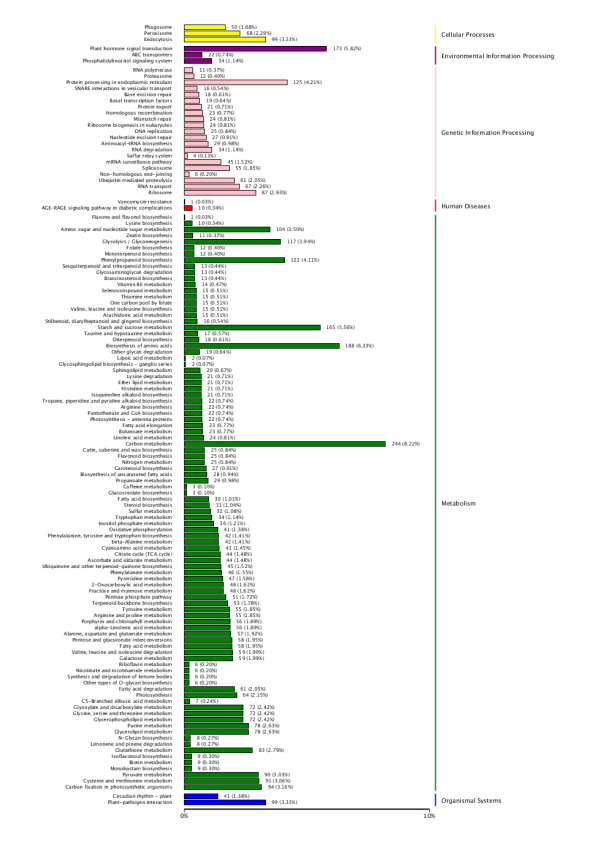

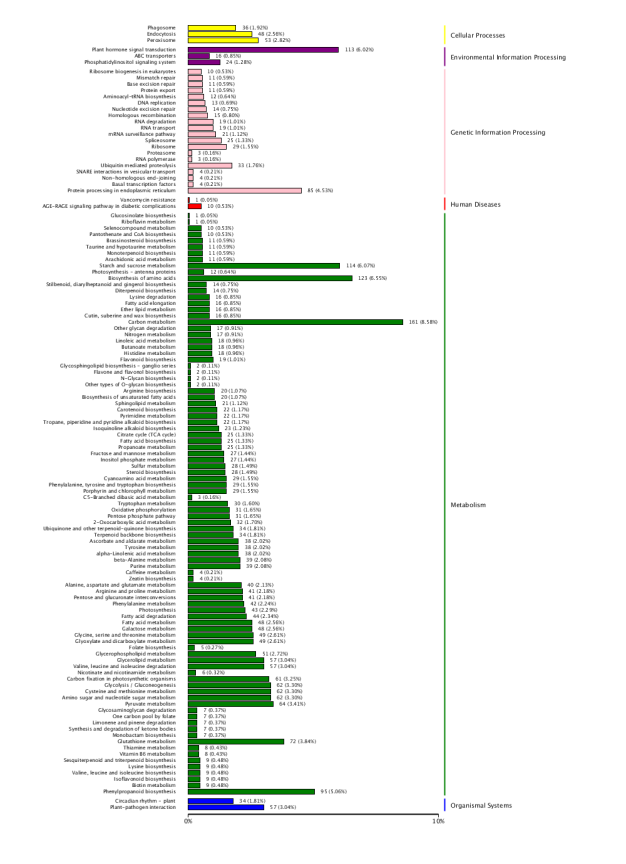


b

a

Figure S1 KEGG notes of differential genes between FH18 and NH5 under drought stress.(a)KEGG notes of differential genes in FH18 compared with CK；(b)KEGG notes of differential genes in NH5 compared with CK
